# Tracking handgrip strength asymmetry from childhood to mid‐life

**DOI:** 10.1111/apa.16930

**Published:** 2023-08-11

**Authors:** Brooklyn J. Fraser, Leigh Blizzard, Grant R. Tomkinson, Ryan McGrath, Terence Dwyer, Alison J. Venn, Costan G. Magnussen

**Affiliations:** ^1^ Menzies Institute for Medical Research University of Tasmania Hobart Tasmania Australia; ^2^ Alliance for Research in Exercise, Nutrition and Activity (ARENA) University of South Australia Adelaide South Australia Australia; ^3^ Healthy Aging North Dakota (HAND) North Dakota State University Fargo North Dakota USA; ^4^ Department of Health, Nutrition, and Exercise Sciences North Dakota State University Fargo North Dakota USA; ^5^ Fargo VA Healthcare System Fargo North Dakota USA; ^6^ The Nuffield Department of Women's & Reproductive Health University of Oxford Oxford UK; ^7^ Murdoch Children's Research Institute Melbourne Victoria Australia; ^8^ Faculty of Medicine, Dentistry and Health Sciences University of Melbourne Melbourne Victoria Australia; ^9^ Baker Heart and Diabetes Institute Melbourne Victoria Australia; ^10^ Research Centre of Applied and Preventive Cardiovascular Medicine University of Turku Turku Finland; ^11^ Centre for Population Health Research University of Turku and Turku University Hospital Turku Finland

**Keywords:** cohort studies, dynamometer, hand strength, prospective studies, upper extremity

## Abstract

**Aim:**

Determine if asymmetric handgrip strength exists in childhood and adulthood and quantify the degree of tracking of handgrip strength asymmetry over time.

**Methods:**

Participants from the Childhood Determinants of Adult Health Study had their right and left handgrip strength measured using handgrip dynamometry in childhood (1985: 9–15 y), young adulthood (2004–06: 26–36 y) and/or mid‐adulthood (2014–19: 36–49 y). Handgrip strength asymmetry was calculated as: strongest handgrip strength/strongest handgrip strength on the other hand. Participants were categorised based on the degree of their asymmetry (0.0%–10.0%, 10.1%–20.0%, 20.1%–30.0%, >30.0%). Tracking was quantified using Spearman's correlations and log binomial regression.

**Results:**

Handgrip strength asymmetry was present in childhood and adulthood (>30.0% asymmetry: childhood = 6%, young adulthood = 3%, mid‐adulthood = 4%). Handgrip strength asymmetry did not track between childhood and young‐ (*r* = 0.06, 95% CI = −0.02, 0.12) and mid‐adulthood (*r* = 0.01, 95% CI = −0.09, 0.10). Tracking was more apparent between young‐ and mid‐adulthood (*r* = 0.16, 95% CI = 0.09, 0.22). Participants with >30.0% asymmetry were at greater risk to maintain this status between childhood and young‐ (RR = 3.53, 95% CI = 1.15, 10.87) and mid‐adulthood (RR = 2.14, 95% CI = 0.45, 10.20).

**Conclusion:**

Although handgrip strength asymmetry tracked relatively poorly, asymmetric handgrip strength was apparent in children and adults. Handgrip strength asymmetry does not exclusively affect older adults and should be considered in protocols to better understand its role across the life course.

**Abbreviations**
ASHFS: Australian Schools Health and Fitness Survey
CDAH: Childhood Determinants of Adult Health
CI: confidence intervals
*r*
: correlation coefficient
RR: relative risk

**Key notes**

- Handgrip strength asymmetry in adults is a risk factor for all‐cause mortality and multimorbidity, but it was unclear if asymmetry exists and persists from early life.
- Handgrip strength asymmetry was present in childhood, young‐ and mid‐adulthood but asymmetric handgrip strength tracked more strongly in adulthood than it did between childhood and adulthood.
- Handgrip asymmetry is not a phenomenon exclusive to older adults, suggesting its potential significance between childhood and mid‐life.

## 1 BACKGROUND

Muscular strength, which is often measured with handgrip strength, is considered a health‐related physical fitness component. Handgrip strength is a powerful biomarker of health^1^, ^2^ and clinical vital sign. Mean handgrip strength has been identified in a large, longitudinal population study of adults as a stronger predictor of all‐cause and cardiovascular mortality than systolic blood pressure.^3^ The handgrip strength test is simple, quick, cost‐effective and a reliable and valid field‐based measure^4^, ^5^ of overall muscular strength.^6^ Standardised handgrip strength test protocols recommend collecting multiple measures with both hands and using the maximal handgrip value for analysis.^7^ Therefore, in addition to measuring maximal handgrip strength, there is potential to improve the predictive value of handgrip dynamometry by additionally assessing handgrip strength asymmetry (i.e. bilateral strength imbalance).

In adults, although handgrip weakness was more strongly associated with premature all‐cause mortality and multimorbidity than handgrip strength asymmetry,^8^, ^9^ asymmetry is an independent risk factor for all‐cause mortality,^8^ multimorbidity,^9^, ^10^ falls,^11^, ^12^, ^13^ lower cognitive function^14^ and functional disability.^15^, ^16^ Handgrip strength asymmetry may reflect a type of muscle dysfunction that precedes declining levels of maximal strength and physical function.^16^ Therefore, screening for asymmetry may help identify those at risk of adverse health outcomes that could have been overlooked by only focussing on weakness.^16^ To better inform clinical screening strategies, it could be important to determine the degree of handgrip strength asymmetry, or tracking, over time. If so, interventions aimed at correcting bilateral strength imbalances and improving long‐term health may be introduced earlier in the lifespan.

Handgrip strength asymmetry has primarily been investigated in middle‐aged and older populations. Therefore, it is unclear how apparent handgrip strength asymmetry is in childhood. By understanding the relationship between maximal handgrip strength and handgrip strength asymmetry in children and determining if handgrip strength asymmetry persists into later life, childhood may emerge as a life stage when strategies aimed at correcting strength imbalances may be best introduced or alternatively, may suggest that handgrip strength asymmetry should be a focus only for those in later life. By using data from a large national cohort followed for over three decades from childhood to mid‐life, this study is uniquely placed to describe the proportion of participants with handgrip strength asymmetry from childhood to mid‐adulthood, and to quantify the degree of tracking of asymmetric handgrip strength across this part of the life course.

## 2 METHODS

### 2.1 Participants

As part of the Australian Schools Health and Fitness Survey (ASHFS), a nationally representative sample of Australian children aged 7–15 years (*n* = 8498) was assessed in 1985. A subset of this cohort aged 9, 12 and 15 years had their right and left handgrip strength measured (*n* = 2798). The Childhood Determinants of Adult Health (CDAH) Study followed these participants into young adulthood (2004–06: 26–36 years of age) and mid‐adulthood (2014–19: 36–49 years of age). At both adult follow‐ups, participants completed pre‐exercise screening and if eligible, had their handgrip strength remeasured. Included in our descriptive analyses of our prospective longitudinal study were participants who had their right and left handgrip strength in childhood (*n* = 2798), young adulthood (*n* = 2117) and/or mid‐adulthood (*n* = 1161). Our tracking analyses included participants who had their handgrip strength measured in both: (a) childhood and young adulthood (*n* = 720); (b) childhood and mid‐adulthood (*n* = 387); or (c) young adulthood and mid‐adulthood (*n* = 859). A flow chart of participation is presented in Figure S1. The ASHFS was approved by the State Directors General of Education. The two adult follow‐up studies were approved by the Southern Tasmania Health and Medical Human Research Ethics Committee and the Tasmania Health and Medical Human Research Ethics Committee. For participation in ASHFS, consent was obtained from a parent and assent obtained from the child and at each follow‐up, participants provided written informed consent.

### 2.2 Maximal handgrip strength

Handgrip strength was measured by maximum voluntary contraction using an isometric handgrip dynamometer (Smedley's Dynamometer, TTM) in childhood, young adulthood and mid‐adulthood. The dynamometer was adjusted to fit the size of the participant's hand. While standing with the dynamometer placed on their opposite shoulder, participants squeezed the dynamometer with maximal effort. Handgrip strength was recorded to the nearest 0.5 kilogram (kg). Participants had one attempt at right and left handgrip strength in childhood and two attempts at both adult time‐points, with the maximum of two attempts retained in analyses. As such, we were unable to investigate the test–retest reliability of handgrip strength asymmetry at each time‐point. Hand dominance (right or left) was self‐reported.

### 2.3 Handgrip strength asymmetry

A handgrip strength asymmetry ratio was calculated as: (maximal handgrip strength of the stronger hand (kg)/maximal handgrip strength of the weaker hand (kg)).^17^ This asymmetry ratio was used to determine the degree of asymmetry, regardless of hand dominance. Participants were categorised into four groups based on the degree of their handgrip strength asymmetry: (1) 0.0%–10.0%; (2) 10.1%–20.0%; (3) 20.1%–30.0%; and (4) >30%.^11^, ^17^ The percent difference in handgrip strength between each hand was quantified as: 100 × (|maximal left handgrip strength − maximal right handgrip strength|)/(overall maximal handgrip strength),^13^ which by including the absolute difference in handgrip strength accounts for the magnitude of asymmetry; and as: 100 × (maximal left handgrip strength − maximal right handgrip strength)/(overall maximal handgrip strength), which by including the signed difference in handgrip strength accounts for the magnitude and direction of asymmetry. All measures of handgrip strength asymmetry were calculated in childhood, young adulthood and mid‐adulthood.

### 2.4 Statistical analyses

All statistical analyses were performed using Stata (v17.0; StataCorp). A summary of the handgrip strength asymmetry ratio, maximal right and left handgrip strength, and the percent difference in bilateral maximal handgrip strength (mean, standard deviation, min, max) and the proportion of participants with handgrip strength asymmetry (number, proportion) were described in childhood, young‐ and mid‐adulthood. Summary values are presented age‐ and sex‐combined and age‐ and sex‐stratified (childhood) and sex‐combined and sex‐stratified (adulthood). If participants had a handgrip strength asymmetry ratio >2.0, maximal handgrip strength values were checked for outliers. One female participant (age = 36 years) with a maximal left handgrip strength value of 77 kg (maximal right handgrip strength = 26 kg) was excluded from analysis as their handgrip strength values were deemed too high for their age and sex in this cohort.

The tracking of the handgrip strength asymmetry ratio and the percent difference in maximal handgrip strength between each hand (continuous measures) between childhood and each adult time‐point and between young‐ and mid‐adulthood was estimated using Spearman's rank‐order correlations (correlation coefficient (*r*) and 95% confidence intervals (CI)). Log binomial regression models (relative risk (RR) and 95% confidence intervals) were used to quantify the tracking of categorical classifications of handgrip strength asymmetry across the life course. All models were adjusted for length of follow‐up, baseline age and sex. Adapting an approach by Seaman et al.,^18^ all analyses (rank correlation, log binomial) included inverse probability weighting with multiple imputation of incomplete baseline data to account for missing data at follow‐up. Results for analyses including inverse probability weighting were not markedly different to complete case analyses. An alpha level of 0.05 was used for all analyses.

## 3 RESULTS

### 3.1 Description of maximal and asymmetric handgrip strength

A summary of maximal and asymmetric handgrip strength at each time‐point are presented in Table 1, with age‐ and/or sex‐stratified summaries presented in Table S1 (childhood) and Table S2 (adulthood). The distribution of maximal and asymmetry handgrip strength at each time‐point is presented in Figure S2, with the age‐related changes presented in Figures S3–S5. The proportion of participants with >30.0% handgrip strength asymmetry was 6% in childhood (*n* = 157), 3% in young adulthood (*n* = 61) and 4% in mid‐adulthood (*n* = 43). In childhood, the proportion of persons with >30.0% handgrip strength asymmetry decreased as age increased, with the proportion for those aged 15 years in childhood most like adult estimates. The mean handgrip strength asymmetry ratio was similar across time‐points, with the percent difference in handgrip strength between hands (accounting for magnitude and direction of asymmetry) greater in adulthood than childhood. At each time‐point, right and dominant handgrip strength were greater on average than left and non‐dominant handgrip strength.

**TABLE 1:** Summary of handgrip strength and handgrip strength asymmetry at each time‐point.

	Childhood (9, 12, 15 years)	Young adulthood (26–36 years)	Mid‐adulthood (36–49 years)
Handgrip strength asymmetry ratio			
N	2798	2117	1160
Mean (SD)	1.11 (0.11)	1.10 (0.09)	1.10 (0.09)
Min	1.00	1.00	1.00
Max	2.54	1.72	1.94
Handgrip strength asymmetry, *n* (%)			
0.0%–10.0%	1577 (56)	1298 (61)	721 (62)
10.1%–20.0%	799 (29)	583 (28)	311 (27)
20.1%–30.0%	265 (10)	175 (8)	85 (7)
>30%	157 (6)	61 (3)	43 (4)
Percent difference in maximal handgrip strength between hands, accounting for magnitude of asymmetry			
Mean (SD)	9.51 (7.65)	8.49 (6.46)	8.45 (6.69)
Min	0.00	0.00	0.00
Max	60.61	41.94	48.54
Percent difference in maximal handgrip strength between hands, accounting for magnitude and direction of asymmetry			
Mean (SD)	−2.57 (11.93)	−4.62 (9.61)	−4.97 (9.57)
Min	−55.81	−41.94	−48.54
Max	60.60	35.19	30.00
Maximal handgrip strength			
Mean (SD)	23.95 (9.02)	39.72 (11.82)	39.08 (11.07)
Min	4.00	11.00	6.00
Max	62.00	78.50	72.50
Maximal right handgrip strength			
Mean (SD)	23.15 (8.94)	38.97 (11.69)	38.39 (11.00)
Min	4.00	10.50	6.00
Max	62.00	78.50	72.50
Maximal left handgrip strength			
Mean (SD)	22,51 (8.72)	37.16 (11.51)	36.50 (10.80)
Min	3.00	9.00	5.00
Max	61.00	69.00	65.00
Dominant hand, *n* (%)			
Left	288 (10)	235 (12)	241 (21)
Right	2506 (90)	1796 (88)	915 (79)
Dominant handgrip strength			
Mean (SD)	23.22 (8.95)	39.06 (11.65)	38.33 (11.04)
Min	4.00	10.50	6.00
Max	62.00	78.50	72,50
Non‐dominant handgrip strength			
Mean (SD)	22.42 (8.70)	37.10 (11.52)	36.59 (10.80)
Min	3.00	9.00	5.00
Max	58.00	72.00	65.00

Abbreviation: SD, standard deviation.

**FIGURE 1:**
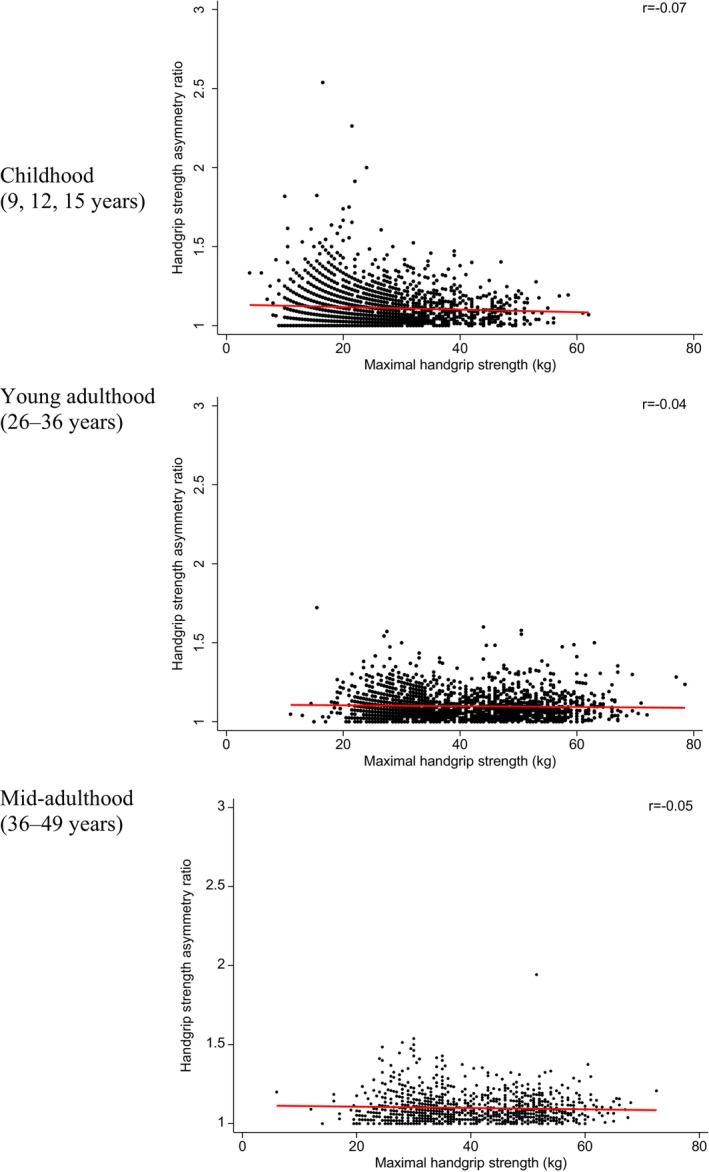
Scatter plot of maximal handgrip strength and handgrip strength asymmetry ratio by time‐point.

Scatter plots between maximal handgrip strength and the asymmetry ratio at each time‐point are presented in Figure 1. All scatter plots showed negligible associations between maximal handgrip strength and handgrip strength asymmetry (childhood: *r* = −0.07, *p* < 0.001; young adulthood: *r* = −0.04, *p* = 0.09; mid‐adulthood: *r* = −0.05, *p* = 0.08). Age‐ and/or sex‐stratified scatter plots are presented in Figure S6 (childhood) and Figure S7 (adulthood). There was no marked difference in the association by age and/or sex in childhood or adulthood.

### 3.2 Tracking of handgrip strength asymmetry

The rank correlation coefficients between handgrip strength asymmetry ratios measured in childhood, young‐ and mid‐adulthood are presented in Table 2. The handgrip strength asymmetry ratio did not appear to track between childhood and each adult time‐point (to young adulthood: *r* = 0.06, 95% CI: −0.02, 0.12; to mid‐adulthood: *r* = 0.01, 95% CI: −0.09, 0.10). Handgrip strength asymmetry ratio tracked relatively weakly between young‐ and mid‐adulthood (*r* = 0.16; 95% CI: 0.09, 0.22). Tracking was markedly stronger when accounting for magnitude and direction of asymmetry (Table S4) than considering magnitude of asymmetry alone (Table S3). Results remained relatively unchanged when analyses were restricted to those participants who provided a measure of handgrip strength asymmetry at all three time‐points (*n* = 282).

**TABLE 2:** Rank correlation between handgrip strength asymmetry ratios calculated at different life stages (childhood, young‐ and mid‐adulthood) adjusted for length of follow‐up, sex and baseline age.

	*n*	Correlation coefficients	95% CI	*p*‐Value
Childhood to young adulthood				
Combined sexes	720	0.06	−0.02, 0.12	0.14
Males	347	0.03	−0.08, 0.13	0.61
Females	373	0.07	−0.04, 0.17	0.20
Childhood to mid‐adulthood				
Combined sexes	387	0.01	−0.09, 0.10	0.92
Males	176	0.05	−0.10, 0.20	0.54
Females	211	−0.03	−0.17, 0.10	0.65
Young‐ to mid‐adulthood				
Combined sexes	859	0.16	0.09, 0.22	3.80e‐06
Males	418	0.14	0.05, 0.24	0.003
Females	441	0.18	0.08, 0.27	1.96e‐04

Abbreviation: CI, confidence intervals.

The relative risk of having different degrees of handgrip strength asymmetry in young‐ and mid‐adulthood are shown in Table 3. Participants with >10% handgrip strength asymmetry in childhood were more likely to maintain this status into young adulthood (RR = 1.14, 95% CI = 0.93, 1.40), compared with children with <10% handgrip strength asymmetry. Similar trends were observed for the tracking of >20% and >30% handgrip strength asymmetry between childhood and each adult time‐point. Handgrip strength asymmetry defined as >10% and >20% tracked between young‐ and mid‐adulthood, with the strongest results observed for participants who maintained >20% handgrip strength asymmetry (RR = 2.95, 95% CI = 1.89, 4.59).

**TABLE 3:** Relative risk of having different degrees of handgrip strength asymmetry at young‐ and mid‐adulthood.

	Unadjusted			Adjusted for sex, baseline age and length of follow‐up.		
	*n*/*N*	RR (95% CI)	*p*‐Value	*n*/*N*	RR (95% CI)	*p*‐Value
>10.0%						
Childhood to young adulthood						
0.0%–10.0%	134/385	1 (REF)			1 (REF)	
>10.0%	132/335	1.15 (0.94, 1.41)	0.16		1.14 (0.93, 1.40)	0.21
Childhood to mid‐adulthood						
0.0%–10.0%	80/208	1 (REF)			1 (REF)	
>10.0%	68/179	0.98 (0.73, 1.31)	0.88		0.95 (0.70, 1.28)	0.72
Young‐ to mid‐adulthood						
0.0%–10.0%	172/521	1 (REF)			1 (REF)	
>10.0%	161/338	1.42 (1.18, 1.71)	2.22e‐04		1.42 (1.18, 1.70)	2.15e‐04
>20.0%						
Childhood to young adulthood						
0.0%–20.0%	56/607	1 (REF)			1 (REF)	
>20.0%	18/113	1.70 (1.00, 2.86)	0.05		1.58 (0.93, 2.67)	0.09
Childhood to mid‐adulthood						
0.0%–20.0%	32/327	1 (REF)			1 (REF)	
>20.0%	12/60	1.52 (0.76, 3.03)	0.24		1.45 (0.73, 2.88)	0.29
Young‐ to mid‐adulthood						
0.0%–20.0%	63/762	1 (REF)			1 (REF)	
>20.0%	25/97	3.00 (1.91, 4.71)	1.90e‐06		2.95 (1.89, 4.59)	1.73e‐06
>30%						
Childhood to young adulthood						
0.0%–30.0%	16/680	1 (REF)			1 (REF)	
>30.0%	4/40	4.34 (1.47, 12.83)	0.01		3.53 (1.15, 10.87)	0.03
Childhood to mid‐adulthood						
0.0%–30.0%	15/373	1 (REF)			1 (REF)	
>30.0%	2/14	2.26 (0.48, 10.59)	0.30		2.14 (0.45, 10.20)	0.34
Young‐ to mid‐adulthood						
0.0%–30.0%	28/834	1 (REF)			1 (REF)	
>30.0%	1/25	0.65 (0.09, 4.85)	0.68		0.84 (0.11, 6.33)	0.87

Abbreviations: CI, confidence intervals; RR, relative risk.

## 4 DISCUSSION

This study was well placed to quantify the tracking of handgrip strength asymmetry between childhood and mid‐life, while also addressing a current knowledge gap by quantifying the proportion of participants with handgrip strength asymmetry in the one cohort at different life stages. Our findings showed that handgrip strength asymmetry was present in childhood, young‐ and mid‐adulthood but asymmetric handgrip strength tracked more strongly in adulthood than it did between childhood and adulthood.

Our results and those from previous studies show that children experience asymmetric handgrip strength, but differences may exist across age groups.^19^, ^20^, ^21^, ^22^ There is currently no consensus on when asymmetry occurs and how apparent it is in early life. Previous studies have suggested that handgrip strength symmetry is observed in children but then may become more asymmetric with increasing age, or that asymmetric handgrip strength may be observed in early childhood and then diminish and reappear as children get older.^20^ Our descriptive results challenge these suggestions by showing that the proportion of participants with >30% asymmetry was greater in childhood than at either adult time‐point, with the proportion of participants with asymmetry decreasing with childhood age. These findings suggest that childhood age could be influencing handgrip strength measurement,^23^ where right and left (or dominant and non‐dominant) handgrip strength mastery may not be as developed in younger children as it is at older ages, for example. However, the handgrip strength test is a reliable and valid measure of musculoskeletal fitness in youth.^4^, ^23^ Therefore, an alternative potential explanation for these findings could be in part due to participants having one attempt at right and left handgrip strength in childhood, and two attempts, with the maximum value used in analyses, at each adult time‐point. A single attempt in childhood may have reduced the ability for children to experience a learned effect and potentially exaggerated the difference in right and left handgrip strength, compared with adulthood. Further, estimates of the proportion of participants with lower degrees of handgrip strength asymmetry (i.e. >10%, 10.1%–20%, 20.1%–30%) were generally similar at all three life stages. To this point, research examining handgrip strength asymmetry has primarily focussed on middle‐to‐older age adults. These findings suggest that handgrip strength asymmetry should not be pigeonholed as a condition that is exclusive to older adults and asymmetric handgrip strength should be considered across the life course beginning in childhood. However, more information on children and young adults is required and additional research should determine if the aetiology of handgrip strength asymmetry differs depending on the life stage at which it is identified. It is plausible that at certain ages, handgrip strength asymmetry may reflect different aetiologies. For example, it may be a risk factor for disease, a consequence of disease, or both, depending on the life stage. Better understanding of what handgrip strength asymmetry represents at different ages (e.g. childhood vs. older adults) will provide more insight into whether it is appropriate to compare proportion estimates.

After showing handgrip strength asymmetry to be present, irrespective of what it reflects, at all examined life‐stages, we then aimed to understand the relationship between maximal and asymmetric handgrip strength and quantify the degree of tracking of handgrip strength asymmetry over time. Our findings suggest that asymmetric handgrip strength tracking is stronger throughout adulthood than between childhood and adulthood. Previous research using CDAH data have shown that age‐ and sex‐standardised measures of maximal right and left handgrip strength not attributable to body mass, track between childhood and young adulthood,^24^ between childhood and mid‐adulthood,^25^ and between young‐ and mid‐adulthood.^25^ When comparing the correlation coefficients for the tracking of maximal right and left handgrip strength in these previous studies and the current results for handgrip strength asymmetry, it is evident that maximal handgrip strength tracks more strongly. Handgrip strength asymmetry also tracks between childhood and adulthood to a lesser degree than other measures such as cardiorespiratory fitness,^26^ blood pressure,^27^ blood lipids^28^ and measures of adiposity.^29^ The weaker tracking observed for handgrip strength asymmetry may suggest that it is more amenable to environmental and lifestyle change, or that it may reflect different aetiologies, have different prognostic utility, underlying pathology and/or risk factor profiles at each life stage.

The marked difference in the tracking of maximal and asymmetric handgrip strength coupled with the negligible correlations between these two measures at all time‐points suggest that maximal and asymmetric handgrip strength are different phenotypes. Drawing direct comparisons between maximal handgrip strength and handgrip strength asymmetry is difficult as maximal handgrip strength is often normalised for different measures of body size. In the literature, however, there is no consensus on the best way to account for body size.^30^ A benefit of measuring and assessing handgrip strength asymmetry is that there is no need to account for a measure of body size, which is appealing when considering and promoting handgrip strength asymmetry as a health‐related measure in future research. Furthermore, the distribution of maximal and asymmetric handgrip strength varied, with the distribution of handgrip strength asymmetry visibly more right skewed. These distributions suggest that maximal handgrip strength may me more amenable to parametric statistical analysis than handgrip strength asymmetry. Future research should examine if cut‐points for asymmetric handgrip strength exists beyond which, one's risk of certain health outcomes increase. Collectively, these findings provide support for the handgrip dynamometer giving rise to two prognostic markers (weakness and strength imbalance). Our study also provided unique perspectives into handgrip strength asymmetry by quantifying the tracking of percent difference in handgrip strength. While both the magnitude and direction of handgrip strength asymmetry fluctuate over time, our findings show the direction of asymmetry to be more stable. These findings suggest that both the direction and magnitude of asymmetry should be considered when analysing handgrip strength asymmetry.

When the degree of handgrip strength asymmetry was categorised into different levels, >20% and >30% asymmetry generally tracked better than >10% asymmetry across all examined time‐points. This is of interest given many reference the “10% rule” when defining handgrip strength asymmetry (i.e. handgrip strength of the dominant hand is generally 10% stronger than the handgrip strength of the non‐dominant hand in adults).^31^, ^32^ More research is needed to better understand if the 10% rule is relevant in children, to define a universal definition of handgrip strength asymmetry, and to understand how different cut‐points of handgrip strength asymmetry influence associations with health outcomes, with our findings providing preliminary support for the >20% or >30% cut‐point, at least in a tracking setting.

The length of time between measures is one of the strongest determinants of how strongly an attribute tracks.^33^ This trend was apparent in our study with the tracking of handgrip strength asymmetry stronger between the shorter adult time‐points than it was between the longer childhood and adulthood time‐points. Although the length of follow‐up is important to consider when interpreting tracking estimates, so too are what lifestyle transitions are occurring between life stages. The weaker tracking between the transition from childhood and adulthood could reflect meaningful life and behavioural changes (e.g. education and occupation changes, parenting and partnering transitions) that may stabilise between young‐ to mid‐adulthood. Future research should aim to identify and compare behavioural and lifestyle correlates of handgrip strength asymmetry at different stages across the life course and investigate factors that influence the tracking of handgrip strength over time, such as changes in lifestyle^34^ and strength between hands (e.g. decreases in the strength of the stronger hand or increases in the strength of the weaker hand).

Potential limitations of this study include loss to follow‐up. To account for missingness and to reduce the likelihood of bias, our analyses included inverse probability weighting. It should also be acknowledged that alternative definitions of handgrip strength asymmetry exist, including those that consider hand dominance. We opted against using these definitions because our sample was not large enough to adequately capture a balanced proportion of participants with right‐ and left‐hand dominance. Furthermore, limited research exists on whether handgrip strength differs between the dominant and non‐dominant hand in children of varying ages and sexes,^19^ and what such differences represent and how meaningful they are (e.g. is it a consequence of behaviour or does it signal an underlying health issue). As such, there is a lack of consensus on whether determining asymmetries by hand dominance are worth examining in younger populations. A standalone measure of absolute difference in handgrip strength could have also been included. Although this approach simplifies asymmetry calculations, it was omitted from our analyses as this measure could lower asymmetry precision with respect to examining handgrip strength on each hand and be influenced by where in the distribution handgrip strength values fall. Instead, percent difference in handgrip strength that included the absolute difference in handgrip strength was examined. Strengths of this study include maximal handgrip strength of participants being measured at all three life stages, over up to 34‐years, among a national sample of Australians.

## 5 CONCLUSION

Despite not tracking strongly into adulthood, our findings indicate a comparable proportion of participants with handgrip strength asymmetry in childhood and adulthood. This highlights that handgrip asymmetry is not a phenomenon exclusive to older age groups, suggesting its potential importance across life. Yet, the implications of these findings call for more comprehensive research, particularly involving children and young adults. This will help to determine the predictive value of handgrip strength asymmetry in these younger populations and to identify the potential health benefits related to symmetric handgrip strength. Further understanding is also necessary to determine whether handgrip strength asymmetry at different life stages reflects similar or different aetiologies. This research is critical not only to establish if handgrip dynamometry provides two independent prognostic markers but also to determine the importance of considering handgrip strength asymmetry alongside maximal handgrip strength in research, health surveillance and promotion.

## FUNDING INFORMATION

The baseline study was supported by grants from the Commonwealth Departments of Sport, Recreation and Tourism, and Health; The National Heart Foundation; and the Commonwealth Schools Commission. The follow‐up studies were funded by grants and fellowships from the National Health and Medical Research Council (211316, 1128373), the National Heart Foundation (GOOH 0578), Veolia Environmental Services and the Mostyn Family Foundation. BJF is supported by a National Heart Foundation of Australia Postdoctoral Fellowship (106588). CGM is supported by a National Health and Medical Research Council (NHMRC) Investigator Grant (APP1176494). Funding bodies and sponsors did not play a role in the study design, collection, analysis and interpretation of data, in the writing of the manuscript, or the decision to submit the manuscript for publication.

## CONFLICT OF INTEREST STATEMENT

The authors declare no conflict of interest.

## Supporting information


Appendix S1
