# Supplementary material for: Tracking handgrip strength asymmetry from childhood to mid‐life
Source: Acta Paediatr. 2023 Aug 11;112(11):2408–17. doi: 10.1111/apa.16930 (PMC10952407; doi:10.1111/apa.16930)
Supplement: Supplementary file 1 — Appendix S1 [file APA-112-2408-s001.docx]

**Online-only supplement**

| Table S1. Summary of handgrip strength and handgrip strength asymmetry in childhood, stratified by age and sex. | | | | | | | |
| --- | --- | --- | --- | --- | --- | --- | --- |
|  |  | Childhood – 9 years | | Childhood – 12 years | | Childhood – 15 years | |
|  |  | Males | Females | Males | Females | Males | Females |
| Handgrip strength asymmetry ratio |  |  | |  | |  | |
|  | N | 478 | 487 | 493 | 489 | 441 | 410 |
|  | Mean (SD) | 1.11 (0.11) | 1.13 (0.12) | 1.11 (0.14) | 1.12 (0.11) | 1.09 (0.08) | 1.12 (0.11) |
|  | Min | 1.00 | 1.00 | 1.00 | 1.00 | 1.00 | 1.00 |
|  | Max | 1.67 | 1.82 | 2.54 | 1.91 | 1.58 | 1.91 |
| Handgrip strength asymmetry, n (%) |  |  |  |  |  |  |  |
|  | 0.0%–10.0% | 273 (57) | 241 (49) | 303 (61) | 257 (53) | 278 (63) | 225 (55) |
|  | 10.1%–20.0% | 136 (28) | 151 (31) | 129 (26) | 140 (29) | 125 (28) | 118 (29) |
|  | 20.1%–30.0% | 44 (9) | 56 (12) | 39 (8) | 57 (12) | 27 (6) | 42 (10) |
|  | >30.0% | 25 (5) | 39 (8) | 22 (4) | 35 (7) | 11 (2) | 25 (6) |
| Percent difference in maximal handgrip strength between hands, accounting for magnitude of asymmetry | |  |  |  |  |  |  |
|  | Mean (SD) | 9.40 (7.56) | 10.74 (8.20) | 8.86 (7.99) | 10.09 (7.88) | 8.10 (6.21) | 9.79 (7.53) |
|  | Min | 0.00 | 0.00 | 0.00 | 0.00 | 0.00 | 0.00 |
|  | Max | 40.00 | 45.16 | 60.61 | 47.73 | 36.84 | 47.73 |
| Percent difference in maximal handgrip strength between hands, accounting for magnitude and direction of asymmetry | |  |  |  |  |  |  |
|  | Mean (SD) | -1.43 (11.98) | -2.09 (13.36) | -1.19 (11.88) | -4.30 (12.06) | -2.05 (10,01) | -4.65 (11.45) |
|  | Min | -40.00 | -45.16 | -55.81 | -47.73 | -36.84 | -35.71 |
|  | Max | 38.46 | 45.00 | 60.61 | 37.93 | 28.72 | 47.73 |
| Maximal handgrip strength |  |  |  |  |  |  |  |
|  | Mean (SD) | 17.11 (3.27) | 15.12 (2.94) | 23.98 (4.96) | 22.62 (4.43) | 38.46 (7.76) | 28.37 (4.54) |
|  | Min | 6.00 | 4.00 | 11.00 | 10.50 | 18.00 | 15.00 |
|  | Max | 27,50 | 24.00 | 47.00 | 42.50 | 62.00 | 41.00 |
| Maximal right handgrip strength |  |  |  |  |  |  |  |
|  | Mean (SD) | 16.44 (3.39) | 14.47 (3.03) | 23.07 (5.10) | 21.99 (4.61) | 37.24 (7.78) | 27.65 (4.69) |
|  | Min | 4.50 | 4.00 | 6.50 | 9.00 | 15.50 | 11.50 |
|  | Max | 27,50 | 24.00 | 47.00 | 42.00 | 62.00 | 41.00 |
| Maximal left handgrip strength |  |  |  |  |  |  |  |
|  | Mean (SD) | 16.16 (3.22) | 14.14 (2.95) | 22.76 (5.03) | 20.99 (4.45) | 36.49 (7.71) | 26.28 (4.57) |
|  | Min | 6.00 | 3.00 | 9.50 | 6.50 | 12.00 | 13.50 |
|  | Max | 25.50 | 22.50 | 46,50 | 42.50 | 61.00 | 40.00 |
| Dominant hand, n (%) |  |  |  |  |  |  |  |
|  | Left | 70 (15) | 47 (10) | 56 (11) | 37 (8) | 39 (9) | 39 (10) |
|  | Right | 408 (85) | 440 (90) | 437 (89) | 452 (92) | 399 (91) | 370 (91) |
| Dominant handgrip strength |  |  |  |  |  |  |  |
|  | Mean (SD) | 16.45 (3.36) | 14,53 (3.03) | 23.18 (5.04) | 22.00 (4.59) | 37.37 (7.80) | 27.86 (4.61) |
|  | Min | 4.50 | 4.00 | 6.50 | 9.00 | 17,50 | 14.50 |
|  | Max | 27.50 | 24.00 | 47.00 | 42.00 | 62.00 | 41.00 |
| Non-dominant handgrip strength |  |  |  |  |  |  |  |
|  | Mean (SD) | 16.15 (3.25) | 14.07 (2.94) | 22.66 (5.09) | 20.98 (4.48) | 36.45 (7.69) | 26.08 (4.59) |
|  | Min | 6.00 | 3.00 | 9.50 | 6.50 | 12.00 | 11.50 |
|  | Max | 25,50 | 22.50 | 46.50 | 42.50 | 58.00 | 40.00 |
| Abbreviations: SD, standard deviation. | | | | | | | |

| Table S2. Summary of handgrip strength and handgrip strength asymmetry in young- and mid-adulthood, stratified by age and sex. | | | | | |
| --- | --- | --- | --- | --- | --- |
|  |  | Young adulthood  (26–36 years) | | Mid-adulthood  (36–49 years) | |
|  |  | Males | Females | Males | Females |
| Handgrip strength asymmetry ratio |  |  | |  | |
|  | N | 1,033 | 1,084 | 554 | 606 |
|  | Mean (SD) | 1.09 (0.08) | 1.10 (0.09) | 1.09 (0.08) | 1.11 (0.10) |
|  | Min | 1.00 | 1.00 | 1.00 | 1.00 |
|  | Max | 1.60 | 1.72 | 1.94 | 1.54 |
| Handgrip strength asymmetry, n (%) |  |  |  |  |  |
|  | 0.0%–10.0% | 667 (65) | 631 (58) | 367 (66) | 354 (58) |
|  | 10.1%–20.0% | 272 (26) | 311 (29) | 141 (26) | 170 (28) |
|  | 20.1%–30.0% | 60 (6) | 115 (11) | 34 (6) | 51 (8) |
|  | >30.0% | 34 (3) | 27 (2) | 12 (2) | 31 (5) |
| Percent difference in maximal handgrip strength between hands, accounting for magnitude of asymmetry | |  |  |  |  |
|  | Mean (SD) | 7.99 (6.30) | 8.97 (6.57) | 7.77 (6.09) | 9.08 (7.15) |
|  | Min | 0.00 | 0.00 | 0.00 | 0.00 |
|  | Max | 37.50 | 41.94 | 48.54 | 35.00 |
| Percent difference in maximal handgrip strength between hands, accounting for magnitude and direction of asymmetry | |  |  |  |  |
|  | Mean (SD) | -3.90 (9.40) | -5.31 (9.77) | -4.25 (8.91) | -5.63 (10.09) |
|  | Min | -37.50 | -41.94 | -48.54 | -35.00 |
|  | Max | 32.77 | 35.19 | 27.27 | 30.00 |
| Maximal handgrip strength |  |  |  |  |  |
|  | Mean (SD) | 49.89 (7.58) | 30.03 (5.07) | 48.69 (7.03) | 30.29 (5.25) |
|  | Min | 13.00 | 11.00 | 26.00 | 6.00 |
|  | Max | 78.50 | 50.00 | 72.50 | 43.50 |
| Maximal right handgrip strength |  |  |  |  |  |
|  | Mean (SD) | 48.88 (7.73) | 29.49 (5.16) | 47.84 (7.21) | 29.77 (5.32) |
|  | Min | 13.00 | 10.50 | 26.00 | 6.00 |
|  | Max | 78.50 | 50.00 | 72.50 | 43.50 |
| Maximal left handgrip strength |  |  |  |  |  |
|  | Mean (SD) | 46.89 (7.69) | 27.89 (5.10) | 45.73 (7.09) | 28.06 (5.30) |
|  | Min | 12.50 | 9.00 | 20.50 | 5.00 |
|  | Max | 69.00 | 46.00 | 65.00 | 42.00 |
| Dominant hand, n (%) |  |  |  |  |  |
|  | Left | 116 (12) | 119 (12) | 112 (20) | 129 (21) |
|  | Right | 881 (88) | 915 (88) | 441 (80) | 474 (79) |
| Dominant handgrip strength |  |  |  |  |  |
|  | Mean (SD) | 48.92 (7.61) | 29.55 (5.13) | 47.82 (7.19) | 29.65 (5.31) |
|  | Min | 13.00 | 10.50 | 20.50 | 6.00 |
|  | Max | 78.50 | 50.00 | 72.50 | 43.50 |
| Non-dominant handgrip strength |  |  |  |  |  |
|  | Mean (SD) | 46.76 (7.73) | 27.76 (5.10) | 45.80 (7.10) | 28.16 (5.35) |
|  | Min | 12.50 | 9.00 | 26.00 | 5.00 |
|  | Max | 72.00 | 46.00 | 65.00 | 42.00 |
| Abbreviations: SD, standard deviation. | | | | | |

| Table S3. Rank correlation between percent difference of handgrip strength between hands (accounting for magnitude of asymmetry) calculated at different life stages (childhood, young- and mid-adulthood) adjusted for length of follow-up, sex, and baseline age. | | | | | |
| --- | --- | --- | --- | --- | --- |
|  | | n | Correlation coefficients | 95% CI | p-value |
| Childhood to young adulthood | |  |  |  |  |
|  | Combined sexes | 720 | 0.06 | -0.02, 0.13 | 0.14 |
|  | Males | 347 | 0.03 | -0.08, 0.13 | 0.61 |
|  | Females | 373 | 0.07 | -0.04, 0.17 | 0.20 |
| Childhood to mid-adulthood | |  |  |  |  |
|  | Combined sexes | 387 | 0.01 | -0.09, 0.10 | 0.92 |
|  | Males | 176 | 0.05 | -0.10, 0.20 | 0.54 |
|  | Females | 211 | -0.03 | -0.17, 0.10 | 0.65 |
| Young- to mid-adulthood | |  |  |  |  |
|  | Combined sexes | 859 | 0.16 | 0.09, 0.22 | 4.04e-06 |
|  | Males | 418 | 0.14 | 0.05, 0.24 | 0.004 |
|  | Females | 441 | 0.18 | 0.08, 0.27 | 1.97e-04 |
| Abbreviations: CI, confidence intervals. | | | | | |

| Table S4. Rank correlation between percent difference of handgrip strength between hands (accounting for magnitude and direction of asymmetry) calculated at different life stages (childhood, young- and mid-adulthood) adjusted for length of follow-up, sex, and baseline age. | | | | | |
| --- | --- | --- | --- | --- | --- |
|  | | n | Correlation coefficients | 95% CI | p-value |
| Childhood to young adulthood | |  |  |  |  |
|  | Combined sexes | 720 | 0.18 | 0.11, 0.26 | 8.32e-07 |
|  | Males | 347 | 0.16 | 0.05, 0.26 | 0.003 |
|  | Females | 373 | 0.22 | 0.12, 0.32 | 1.50e-05 |
| Childhood to mid-adulthood | |  |  |  |  |
|  | Combined sexes | 387 | 0.18 | 0.08, 0.28 | 3.81e-04 |
|  | Males | 176 | 0.17 | 0.02, 0.31 | 0.03 |
|  | Females | 211 | 0.19 | 0.05, 0.33 | 0.01 |
| Young- to mid-adulthood | |  |  |  |  |
|  | Combined sexes | 859 | 0.33 | 0.26, 0.39 | 7.86e-23 |
|  | Males | 418 | 0.30 | 0.20, 0.39 | 6.70e-10 |
|  | Females | 441 | 0.37 | 0.28, 0.45 | 2.04e-15 |
| Abbreviations: CI, confidence intervals. | | | | |  |


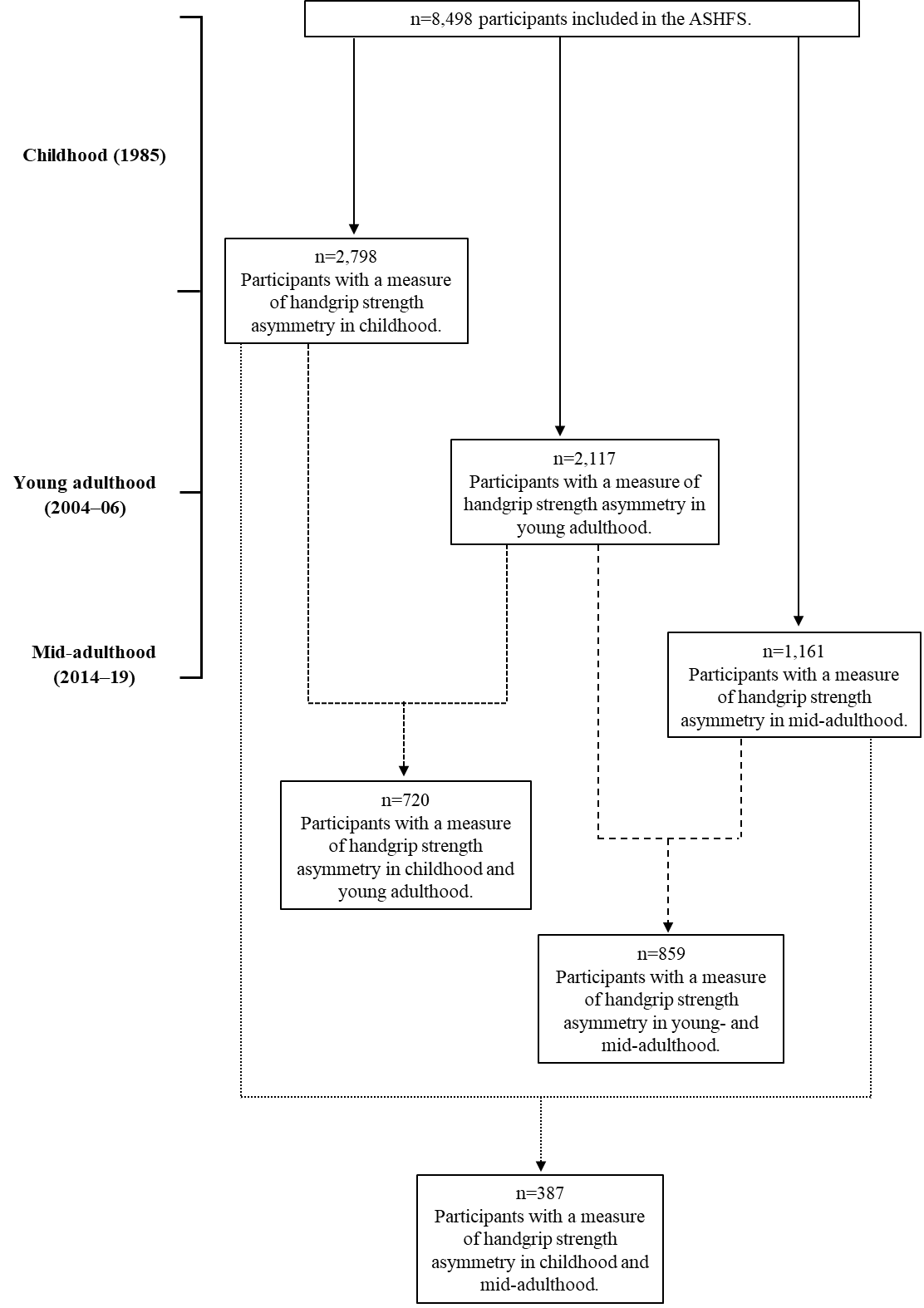


Figure S1. Flow chart of participation.

Abbreviations: ASHFS, Australian Schools Health and Fitness Survey.

|  | Handgrip strength asymmetry ratio | Maximal handgrip strength |
| --- | --- | --- |
| Childhood (9, 12, 15 years) |  |  |
| Young adulthood (26–36 years) |  |  |
| Mid-adulthood (36–49 years) |  |  |

Figure S2. The distribution of handgrip strength asymmetry ratio and maximal handgrip strength at each time-point.

|  | Handgrip strength asymmetry ratio | Maximal handgrip strength |
| --- | --- | --- |
| Childhood (9, 12, 15 years) |  |  |
| Young adulthood (26–36 years) |  |  |
| Mid-adulthood (36–49 years) |  |  |

Figure S3. Scatter plot of handgrip strength asymmetry ratio, maximal handgrip strength and age at each time-point.

|  | Males | Females |
| --- | --- | --- |
| Childhood (9, 12, 15 years) |  |  |
| Young adulthood (26–36 years) |  |  |
| Mid-adulthood (36–49 years) |  |  |

Figure S4. Scatter plot of handgrip strength asymmetry ratio and age at each time-point, stratified by sex.

|  | Males | Females |
| --- | --- | --- |
| Childhood (9, 12, 15 years) |  |  |
| Young adulthood (26–36 years) |  |  |
| Mid-adulthood (36–49 years) |  |  |

Figure S5. Scatter plot of maximal handgrip strength and age at each time-point.

|  | Males | Females |
| --- | --- | --- |
| Childhood – 9 years |  |  |
| Childhood – 12 years |  |  |
| Childhood – 15 years |  |  |

Figure S6. Scatter plot of maximal handgrip strength and handgrip strength asymmetry ratio in childhood, stratified by age and sex.

|  | Males | Females |
| --- | --- | --- |
| Young adulthood  (26–36 years) |  |  |
| Mid-adulthood  (36–49 years) |  |  |

Figure S7. Scatter plot of maximal handgrip strength and handgrip strength asymmetry ratio in young- and mid-adulthood, stratified by sex.
